# Identification and enhancing production of a novel macrolide compound in engineered *Streptomyces peucetius*†

**DOI:** 10.1039/d0ra06099b

**Published:** 2021-01-14

**Authors:** Van Thuy Thi Pham, Hue Thi Nguyen, Chung Thanh Nguyen, Ye Seul Choi, Dipesh Dhakal, Tae-Su Kim, Hye Jin Jung, Tokutaro Yamaguchi, Jae Kyung Sohng

**Affiliations:** Department of Life Science and Biochemical Engineering, SunMoon University 70 Sunmoon-ro 221, Tangjeong-myeon Asan-si Chungnam 31460 Republic of Korea jksohng@sunmoon.ac.kr; Department of Pharmaceutical Engineering and Biotechnology, SunMoon University 70 Sunmoon-ro 221, Tangjeong-myeon Asan-si Chungnam 31460 Republic of Korea

## Abstract

*Streptomyces peucetius* produces doxorubicin and daunorubicin, which are important anticancer drugs. In this study, we activate peucemycin, a new antibacterial compound, using an OSMAC strategy. In general, bioactive compounds are produced in a higher amount at room temperature; however, in this study, we have demonstrated that a bioactive novel compound was successfully activated at a low temperature (18 °C) in *S. peucetius* DM07. Through LC-MS/MS, IR spectroscopy, and NMR analysis, we identified the structure of this compound as a γ-pyrone macrolide. This compound was found to be novel, thus named peucemycin. It is an unusual 14-membered macrocyclic γ-pyrone ring with cyclization. Also, peucemycin exhibits potential antibacterial activity and a suppressive effect on the viability of various cancer cell lines.

## Abstract 2

Activation of peucemycin in *S. peucetius* DM07 by the OSMAC strategy.

## Graphical abstract

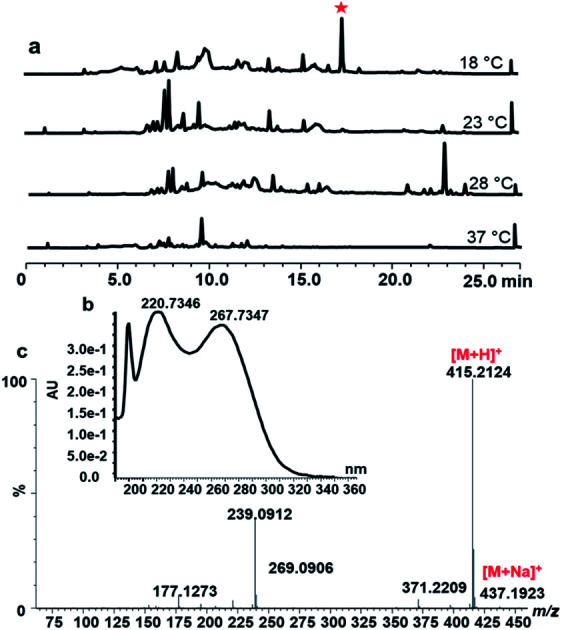

## Introduction

*Streptomyces* are Gram-positive filamentous bacteria with the ability to produce a wide range of secondary metabolites, many of them are antimicrobial or anticancer drugs.^1–3^ The analysis of the genome sequences of *Streptomyces* using bioinformatics tools such as antiSMASH^4^ indicates that they possess more than 20 biosynthesis gene clusters (BGC).^5^ However, most of these BGCs are either expressed or not expressed under laboratory conditions. Therefore, several approaches have been utilized to activate these silent BGCs and/or increase the titer of production to characterize the encoded compounds; these approaches include either culture conditioning or genetic manipulation.^6–8^

One strain many compound (OSMAC) is a simple and powerful approach used to activate numerous compounds such as penicibrocazines A–I, and brocaryrrozins A-B.^9,10^ Many successful OSMAC approaches have been reported to produce new compounds using various media,^11,12^ salinity,^13^ and metal ions.^14^ In addition, we applied the OSMAC approach by varying the culture conditions as temperature is a very important factor in the activation of specific compounds.^15,16^

*Streptomyces peucetius* ATCC 27952 is known for producing important anthracyclines: daunorubicin (DNR) and doxorubicin (DXR).^17^ Several secondary metabolites have been explored through mass and NMR analyses; for example, 1,3,6,8-tetrahydroxynaphthalene, peucechelin, hopene, and geosmin.^18–21^ The *in silico* analysis of the *S. peucetius* genome exhibited that 68 BGGs exist in this strain including diverse metabolites such as PKS, NRPS, terpenes, and siderophores.^22^

Structure motif γ-pyrones have biologically active compounds.^23^ For example, candelalide A, B, C from *Sesquicillium candelabrum* play a role in a blocker of the voltage-gated potassium channel Kv1.3;^24^ aureothin from *Streptomyces thioluteus* has numerous biological assays and exhibits antifungal and antibacterial action;^25^ actinopyrone A, B, C from *Streptomyces pactum* has microbial activity;^26^ and prenylflavones, prenylflavonoids and xanthone psorospermin exhibit cytotoxic activity.^27,28^ Macrolides are a 14- to 16-membered lactone ring, such as erythromycin, azithromycin, and tylosin, and are used as an antibiotic in veterinary medicine.^29^ Macrolides are important in human therapeutics as they are safe to use in β-lactam-allergic patients, period pregnancy, pediatric and elderly patients.^30^

Herein, we applied the OSMAC approach by changing the culture conditions as temperature leads to the activation of a novel compound in *S. peucetius*; the isolation and structure of which were elicited *via* LC-MS/MS and NMR analyses. Furthermore, we examined the biological activity of the novel compound, which shows antibacterial and anticancer activity.

## Materials and methods

### Bacterial strains, medium and growth conditions

*S. peucetius* DM07 was used for this experiment. *S. peucetius* DM07 was a disrupted doxorubicin biosynthetic gene cluster from *S. peucetius* ATCC 27952 (accession number CP022438.1) by Singh *et al.*^21^ The seed culture was cultivated in a 50 mL R2YE medium, shaking at 200 rpm in an Erlenmeyer flask at 28 °C for 48 h.^31^ To produce secondary metabolite, *S. peucetitus* DM07 was cultured in a 50 mL NDYE medium (maltose 22.5 g L^−1^, yeast extract 5.6 g L^−1^, NaNO_3_ 4.28 g L^−1^, K_2_HPO_4_ 0.23 g L^−1^, HEPES 4.77 g L^−1^, MgSO_4_·7H_2_O 0.12 g L^−1^, NaOH 0.4 g L^−1^, and 2 mL of the trace element solution, pH 7.2) and incubated in a shaking incubator at 200 rpm at various temperatures. For evaluating the effect of temperature we first selected 37 °C (optimal temperature for few actinobacteria) and 28 °C (most suitable for *S. peucetius* for the growth and production of secondary metabolites such as doxorubicin and doxorubicin). Then, we created a gradient of temperature with 5 °C difference at a lower scale such as 23 °C, 18 °C and 13 °C, and 5 °C difference at a higher scale as 43 °C and 48 °C (data excluded in the analysis because no growth was observed in temperature above 37 °C and below 18 °C).

### Fermentation, extraction, and analysis of novel polyketide production

*S. peucetius* DM07 strain was cultivated in the 50 mL NDYE medium *via* continuous shaking at 200 rpm at various temperatures for 72 h. After fermentation, 100 mL of ethyl acetate was added to extract compounds from the culture broth. Next, 1 mL of methanol was dissolved in the evaporating ethyl acetate of the extract. The resultant methanol extract was analyzed by a Thermo HPLC series 1100 with a Thermo-C_18_ column (5 μm, 4.6 × 250 mm). The column was equilibrated with 100% solvent A 0.1% trifluoroacetic acid (TFA) in the water and 0% solvent B (acetonitrile (ACN)), and was then adjusted following a linear gradient (1–20 min, from 0% B to 100% B, 20–23 min, 100% B, 23–25 min, from 100% B to 0% B, 25–27 min, 0% B) at a flow rate of 1 mL min^−1^ and with UV detection at 268 nm.

The *S. peucetius* DM07 strain was fermented at 18 °C for 72 h in a fermenter. The fermentation broth (5 L) was extracted with 10 L of ethyl acetate and evaporated under a reduced pressure. After that, the extract was dissolved in 50 mL methanol. The crude extract was used for mass analysis and in the purification process.

The mass analysis was performed *via* reversed-phase chromatography ultra-high-performance liquid chromatography electrospray ionization quadrupole time of flight high-resolution mass spectrometry (UPLC-ESI-Q-TOF-HRMS). Samples were eluted with a gradient solvent mobile phase of 0.1% TFA in water and acetonitrile (0 to 12 min) at 35 °C. The 10 μL of the sample was injected. An LC-MS analysis was performed on a high-resolution mass spectrometer equipped with an electrospray ionization source with 3 kV, 300 °C, and 600 L h^−1^ as the capillary voltage, desolvation gas temperature, and flow rate, respectively.

The extract of the culture broth was examined using an ultimate 3000 UPLC (Thermo Fisher Scientific) with a C_18_ column (YMC-Pack ODS-AQ, 150 × 20 mm^2^) connected to a UV detector (197.7, 220.7, and 267.7 nm). The parameters used were binary gradient: 100% water (solvent A); 100% acetonitrile (solvent B); 0% B (0–3 min), 0% B to 10% B (linear gradient, 3–5 min), 10% B to 45% B (linear gradient, 5–10 min), 45% B to 75% B (linear gradient, 10–28 min), 75% B to 100% B (linear gradient, 28–32 min) 100%, 100% B to 0% B (32–35 min). The flow rate to purify the pure compound was 10 mL min^−1^.

The purified compound was dried, lyophilized, dissolved in dimethyl sulfoxide (DMSO-*d*_6_), and accessed in a 700 MHz spectrometer using a Bruker BioSpin nuclear magnetic resonance (NMR) spectrometer (Billerica, USA) for analyses, including one-dimensional (1D) ^1^H-nuclear magnetic resonance (NMR),^13^C-NMR, and two-dimensional (2D) correlation spectroscopy (COSY), nuclear Overhauser effect spectroscopy (NOESY), rotational frame NOE spectroscopy (ROESY), heteronuclear single quantum correlation (HSQC) and heteronuclear multiple bond correlation (HMBC) analyses (Ochang, Republic of Korea). IR (Infrared Spectroscopy) spectra were recorded using a Bruker VERTEX 70 FTIR spectrometer using the analysis function group.

### Biological assays

The antibacterial activity of peucemycin was investigated on a petri dish by the disc-diffusion method^32^ against Gram-positive bacteria as *Bacillus subtilis* ATCC 6633, *Kocuria rhizophila* NBRC 12708, *Micrococcus luteus* KACC 13377, *Staphylococcus aureus* CCARM 3634 (MRSA), and Gram-negative bacteria as *Klebsiella pneumoniae* subsp. *pneumoniae* ATCC 10031, *Proteus hauseri* NBRC 3851, and *Salmonella enterica* ATCC 14028. Peucemycin was added to paper discs in various amounts (5.175, 10.35, 20.7, 41.4, and 82.8 μg per disc). Positive control erythromycin was added (73.3 μg per disc). The test compounds were dissolved in DMSO, and 2 μL of DMSO was added to a paper disc as a negative control. The plates were incubated at 37 °C for 16–24 h.^33^

Peucemycin was also evaluated for its anticancer activity against six types of cancer cell lines. Human cancer cell lines HeLa, A549, Hep3B, A375SM, U87MG, and AGS, and a human lung fibroblast cell line MRC-5 were purchased from the Korean Cell Line Bank (KCLB, Seoul, Korea). AGS gastric cancer cells were grown in an RPMI 1640 medium by adding 10% fetal bovine serum (FBS; Invitrogen). HeLa cervical cancer cells, Hep3B liver cancer cells, A375SM skin cancer cells, A549 lung cancer cells and MRC-5 lung normal cells were maintained in a Dulbecco's modified Eagle's medium (DMEM; Invitrogen, Grand Island, NY, USA) containing 10% FBS. U87MG brains cancer cells were cultured in a minimum essential medium (MEM) supplemented with 10% FBS. All cells were incubated at 37 °C in a humidified 5% CO_2_ incubator. For the cell viability assay, various cancer cells were plated at a 2 × 10^3^ cells per well density in 96-well culture plates. Compound peucemycin was added to each well in various concentrations (0–400 μM) and maintained at 37 °C for 72 h. A 3-(4,5-dimethylthiazol-2-yl)-2,5-diphenyltetrazolium bromide (MTT) colorimetric assay was performed to check cell viability. Briefly, 50 μL of MTT (2 mg mL^−1^ stock solution) was added to the plates and maintained at 37 °C for 4 h. Each well was removed from the medium and then 100 μL of dimethyl sulfoxide was added. A microplate spectrophotometer was used to measure the absorbance wavelength at 540 nm (Thermo Scientific Multiskan® Spectrum). The cytotoxicity assays were executed on triplets, and the results are shown as mean values standard error (SE).

## Results

### Activation of peucemycin in *S. peucetius* DM07 by the OSMAC strategy

**Fig. 1:**
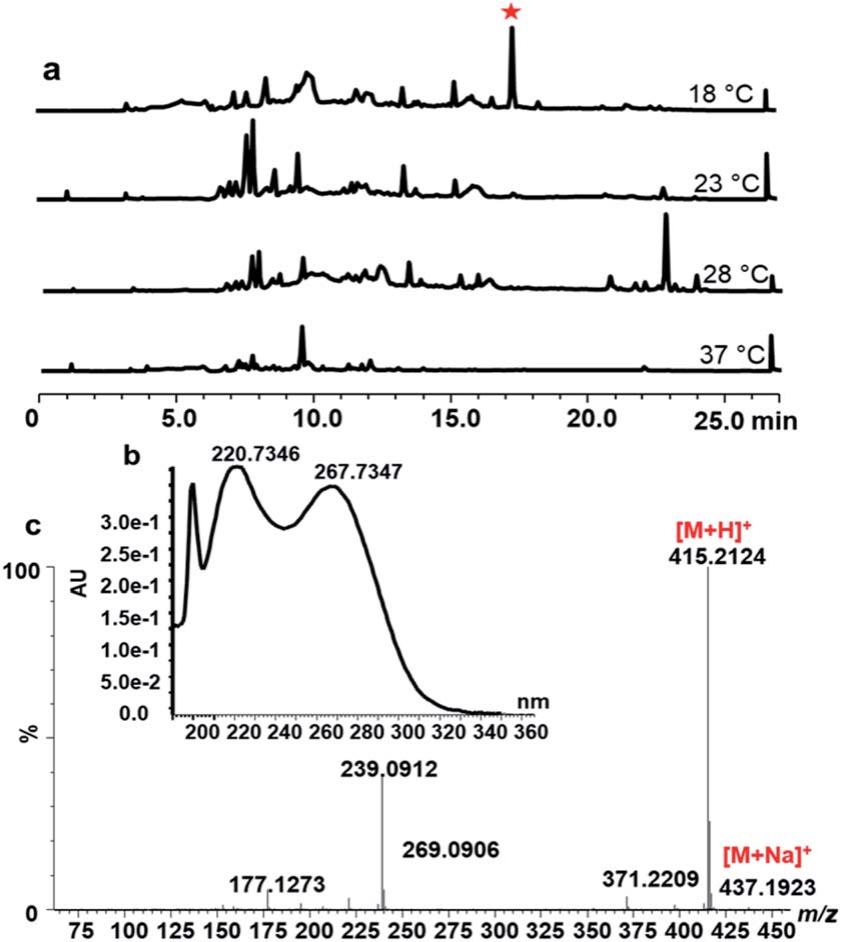
Activation and analysis of peucemycin in *S. peucetius* DM07 (a) HPLC analysis on the metabolite profile of *S. peucetius* DM07 at different temperatures: 18 °C, 23 °C, 28 °C and 37 °C for 72 h. The profile shows that new compound is detected at 17.5 min (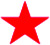 mark) when *S. peucetius* DM07 was cultured at 18 °C. (b) UV absorption of peucemycin. (c) HRMS spectrum of the new peak (peucemycin), the component generates a [M + H]^+^ ion at *m*/*z* 415.21.

The strain *S. peucetius* DM07 was cultured for 72 h in the NDYE medium at various temperatures: 18 °C, 23 °C, 28 °C and 37 °C. The secondary metabolite of *S. peucetius* DM07 was extracted by ethyl acetate and analysed by HPLC and LC-MS. When comparing secondary metabolites at different temperatures, a new peak appeared at 17.5 min with the 18 °C culture conditions, while this peak was absent at other temperatures (Fig. 1a). The peak shows UV spectra absorption maxima at 199.73, 200.73, and 267.73 nm (Fig. 1b). The compound was isolated and purified from 5 L fermentation at 18 °C.

### Structure elucidation of peucemycin

**Fig. 2:**
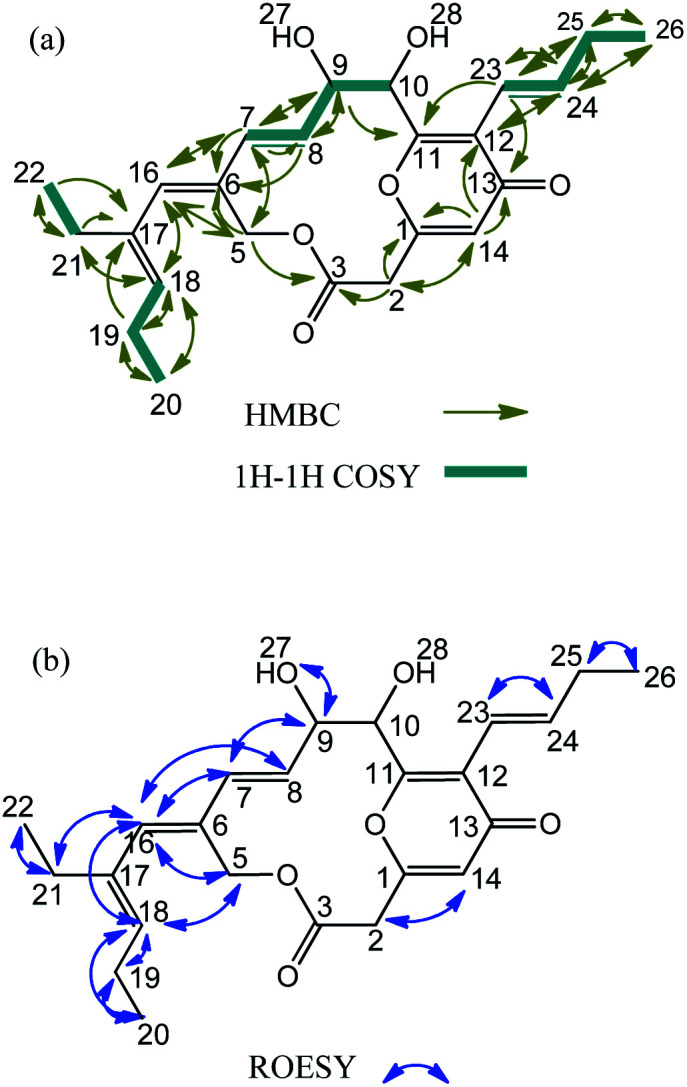
(a) The correlation of ^1^H–^1^H COSY and HMBC of peucemycin. (b) The correlation of ^1^H–^1^H COSY and HMBC of peucemycin. The arrows denote the decoupling experiment. The head arrows denote signal effected. The green line denotes the correlation between hydrogens.

The compound was obtained as a yellow powder that is soluble in DMSO, and its structure was investigated by NMR and was found to correspond to a 14-membered macrocyclic compound containing a γ-pyrone ring (Table 1, Fig. 2 and S2†). The mass fragmentation analysis indicates that the ion fragments correspond to [M + H]^+^ 415.21 *m*/*z*, (M − C)^+^ 177.12 *m*/*z*, (M − A + 2H)^+^ 239.09 *m*/*z*, (B)^+^ 221.08 *m*/*z* (Fig. 1c and S1b†). The molecular formula of the compound was established to be C_24_H_30_O_6_ with *m*/*z* values 414.

**Table 1:** ^1^H- and ^13^C-NMR data of peucemycin in DMSO-*d*_6_^a^

No.	*δ*_C_, type	*δ*_H_ (*J*, Hz)	No.	*δ*_C_, type	*δ*_H_ (*J*, Hz)
1	160.46, C		14	114.78, CH	6.21 (1H, d, 0.96)
2	39.71, CH_2_	3.66 (1H, dd, 15.8, 1.16), 3.73 (1H, d, 15.88)	16	136.88, CH	6.08 (1H, s)
3	167.60, CO		17	137.30, C	
5	60.99, CH_2_	4.97 (1H, d, 12.41), 5.02 (1H, d, 12.42)	18	132.63, CH	5.18 (1H, td, *J* = 7.40, 7.39, 1.37)
6	132.92, C		19	21.24, CH_2_	2.10 (2H, m)
7	128.71, CH	6.01 (1H, dd, 16.07, 1.61)	20	14.78, CH_3_	0.97 (3H, t, 7.5)
8	130.71, CH	5.70 (1H, dd, 16.19, 4.91)	21	23.71, CH_2_	2.08 (2H, m)
9	74.86, CH	4.32 (1H, q, 4.65, 4.65, 4.33)	22	13.71, CH_3_	0.91 (3H, t, 7.6)
10	72.16, CH	4.91 (1H, s)	23	119.22, CH	6.29 (1H, d, 15.72)
11	163.22, C		24	138.74, CH	6.83 (1H, dt, 15.79, 6.59, 6.59)
12	122.70, C		25	27.08, CH_2_	2.15 (2H, td, 7.07, 6.88, 1.58)
13	178.55, CO		26	14.01, CH_3_	1.02 (3H, t, 7.5)

a Spectra recorded at 700 MHz (^1^H) and 176 MHz (^13^C).

The IR spectrum of the compound showed absorption bands for the carbonyl stretch C=O of the aliphatic esters that appear at 1738 cm^−1^, a C–O stretch appears at 1287 cm^−1^, a stretching vibration of the C=C bond (olefins) at 1653 cm^−1^, a CH_3_-asymmetric stretching-vibration absorption at 2972 cm^−1^, a CH_2_-stretching-vibration absorption at 2936 cm^−1^, a CH-asymmetric stretching-vibration absorption at 2862 cm^−1^, and the carboxylic acid with –OH stretching absorption from 3522 to 3192 cm^−1^ (Fig. S1†).^34^

The ^1^H and ^13^C NMR data of the compound in DMSO-*d*_6_ is summarized in Table 1. The ^1^H–^13^C connectivity of all bonds was confirmed by an HMQC. The COSY and HMBC spectra are shown in Fig. 2a. A comprehensive analysis of 1D and 2D NMR spectra reveals the ^1^H-NMR spectrum of peucemycin: three methyl groups at *δ*_H_ 0.91 (t, *J* = 7.6 Hz, 3H), *δ*_H_ 0.97 (t, *J* = 7.5 Hz, 3H), *δ*_H_ 1.02 (t, *J* = 7.5 Hz, 3H); two singlets *δ*_H_ 4.91 (s, 1H), and 6.08 (s, 1H); five doublets at *δ*_H_ 3.73 (d, *J* = 15.88 Hz, 1H), 4.97 (d, *J* = 12.41 Hz, 1H), 5.02 (d, *J* = 12.42 Hz, 1H), 6.21 (d, *J* = 0.96 Hz, 1H), 6.29 (d, *J* = 15.72 Hz, 1H); three double of doublets *δ*_H_ 3.66 (dd, *J* = 15.8, 1.16 Hz, 1H), 6.01 (dd, *J* = 16.07, 1.61 Hz, 1H), 5.70 (dd, *J* = 16.19, 4.91 Hz, 1H); two triple of doublets *δ*_H_ 5.18 (td, *J* = 1.37, 7.39, 7.40 Hz, 1H), and 2.15 (td, *J* = 1.58, 7.07, 6.88 Hz, 2H); one double of triplets *δ*_H_ 6.83 (dt, *J* = 6.59, 6.59, 15.79 Hz, 1H); one quartet *δ*_H_ 4.32 (q, *J* = 4.65, 4.65, 4.33 Hz, 1H); and multiplets 2.12–2.06 (m, 4H) (Fig. S2a†). The ^13^C-NMR spectrum of peucemycin revealed twenty-four carbon signals. The ^13^C-NMR spectrum of peucemycin revealed twenty-four carbon signals in combination with DEPT and HSQC spectra, and these can be categorized as conjugated ketone carbonyls at *δ*_C_ 178.55 (C-13), an ester carbonyl group at *δ*_C_ 167.6 (C-3), oxy-quaternary sp^2^ at *δ*_C_ 160.46 (C-1), 163.22 (C-11), oxy-methine at *δ*_C_ 74.86 (C-9), 72.16 (C-10), quaternary sp^2^*δ*_C_ 132.92 (C-6), 122.7 (C-12), 137.30 (C-17), methine sp^2^*δ*_C_ 128.71 (C-7), 130.71 (C-8), 114.78 (C-14), 136.88 (C-16), 132.63 (C-18), 119.22 (C-23), 138.74 (C-24), methylene *δ*_C_ 39.71 (C-2), 60.99 (C-5), 21.24 (C-19), 23.71 (C-21), 27.08 (C-25) and three methyl *δ*_C_ 14.78 (C-20), 13.71 (C-22), 14.01 (C-26) carbon (Fig. S2b and e†).

The consecutive COSY correlation observed between H-7/H-8, H-23/24, H-19/Me-20, H-21/Me-22 and H-25/Me-26 together with the HMBC correlations from Me-20 to C-19, 18, Me-22 to C-21, C-17 and Me-26 to C-25, C-24 indicate the presence of three methyl groups at C-20, C-22 and C-26 (Fig. 2c). The COSY correlations between H-7/H-8, H-23/H-24 along with the HMBC correlations from H-7 and H-8 to C-9, C-6 and from H-23, and H-24 to C-25 confirm the presence of a methane group (Fig. 2a). H-14 showed HMBC correlation to C-2, C-1, C-13 and C-12, suggesting the presence of a ring (Fig. 2a and S2f†). The relative configuration of peucemycin was deduced through the analysis of its ROESY spectrum. The ROE correlations between H-2/H-14, H-10/H-23, H-7/H-9, H-7/H-16, H-8/H-5, H-5/H-16, H-16/H-18, H-16/H-21, Me-20/H-19, Me-22/H-21, Me-26/H-25, H-9/OH-27 suggest that these protons are on the same side of the molecule. The ROE correlations between H-27/H-28 showed negative signals, which indicate that OH-27 and OH-28 are attached syn-addition confirmation (Fig. 2b and S2d†).

Based on the mass spectrometry and NMR spectroscopy, the compound was named as (6*Z*,7*E*)-12-((*E*)-but-1-en-1-yl)-6-((*E*)-2-ethylpent-2-en-1-ylidene)-9,10-dihydroxy-4,15-dioxabicyclo[9.3.1]pentadeca-1(14),7,11-triene-3,13-dione (peucemycin). The chemical structure of peucemycin shows a novel chemical architecture, thus it was allocated as a novel compound.

### Biological activity

**Fig. 3:**
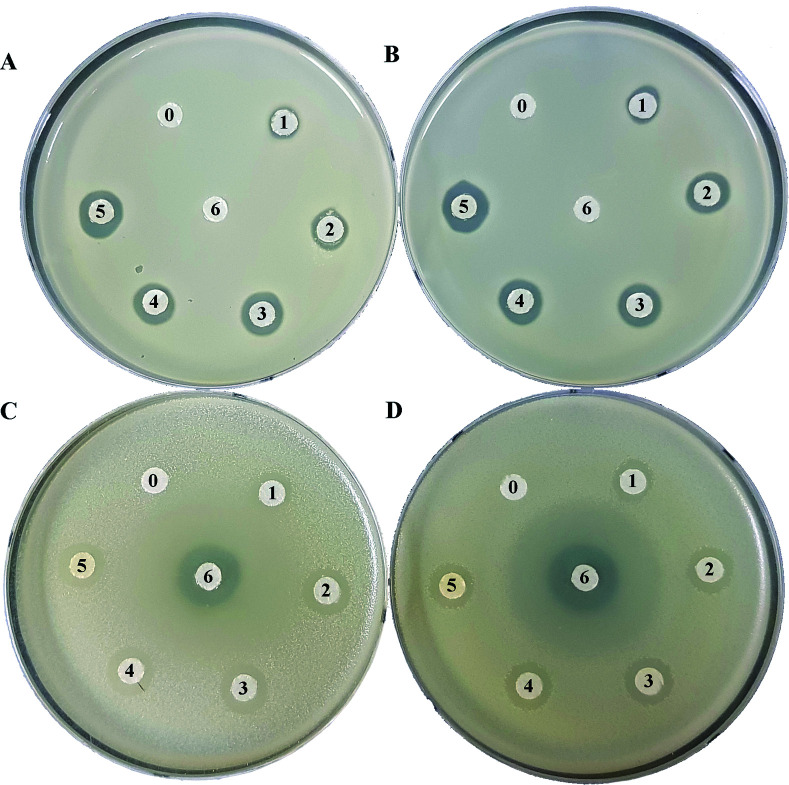
Evaluation of the antibacterial activity of peucemycin against different microbial pathogens using the disc-diffusion assay. (A) *Staphylococcus aureus* CCARM 3634 (MRSA), (B) *Micrococcus luteus* KACC 13377, (C) *Sabmorella enteria* ATCC 14028, and (D) *Proteus hauseri* NBRC 3851 were cultivated on MHA discs and peucemycin was loaded at different concentrations (0) negative control DMSO (1) 5.175 μg (2) 10.35 μg (3) 20.7 μg, (4) 41.4 μg, and (5) 82.8 μg, (6) erythromycin standard was loaded at a concentration of 7.33 μg mL^−1^. The diameter of the zone of inhibition was measured to determine the antibacterial potency of compound.

The antibacterial activity for peucemycin showed that it does not show activity against *B. subtilis*, *K. rhizophila* and *K. pneumoniae* (data not show) but it exhibits antibacterial activity against Gram-positive bacteria (*M. luteus* and *S. aureus*) and Gram-negative bacteria (*P. hauseri* and *S. enterica*). It was observed that the compound exhibits antibacterial potency at different concentrations: 5.175, 10.35, 20.7, 41.4, and 82.8 μg per disc, whereas the standard erythromycin (7.33 μg per disc) was used as the positive control and DMSO was used as the negative control (Fig. 3). The results showed that erythromycin exhibits bioactivity towards *S. enteria* and *P. hauseri* but does not affect the *S. aureus* and *M. luteus* growth, while peucemycin exhibits activity against all the 4 pathogens. Peucemycin showed bactericidal activity towards *S. aureus* and *M. luteus* and bacteriostatic activity towards *S. enteria* and *P. hauseri*.

**Fig. 4:**
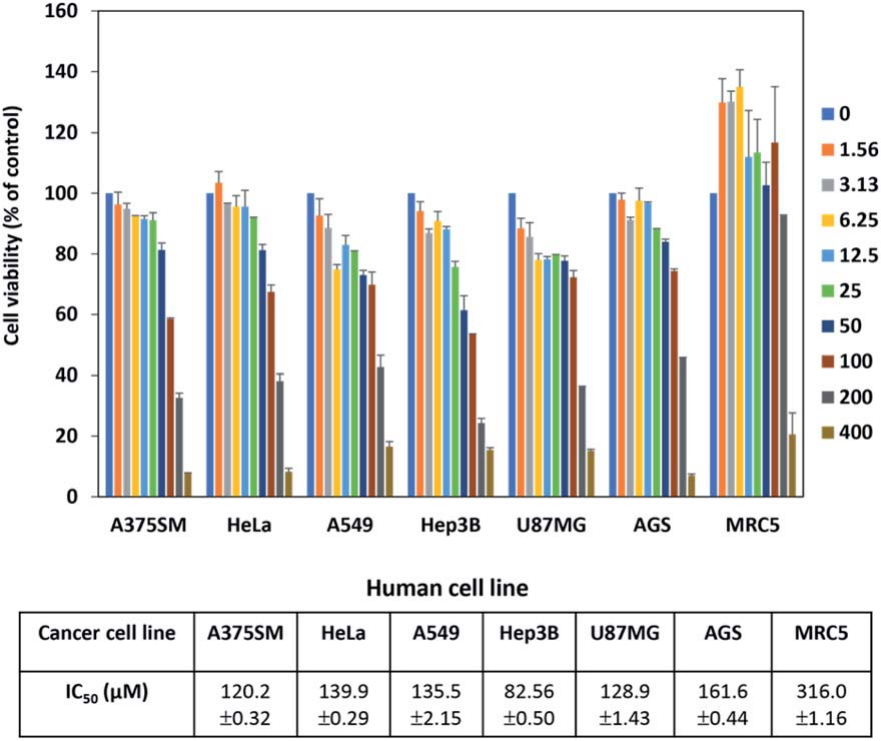
Anticancer activities of peucemycin with various cancer cell lines.

In addition, *S. peucetius* is a prominent producer of anticancer compounds, such as doxorubicin. Hence, we were interested to evaluate the anticancer activity of peucemycin; therefore, we checked the cell inhibitory effect of peucemycin by the assessment of the viability of various tumor cell lines. Although the effective dose for anticancer is high, peucemycin has a suppressive effect on the viability of various cancer cell lines. The IC_50_ value of peucemycin against cancer cells for AGS cells was at 161.6 μM, HeLa cells at 139.9 μM, A549 cells at 135.5 μM, U87MG at 128.9 μM, A375SM cells at 120.2 μM, and Hep3B at 82.56 μM. The compound exhibited the highest growth-inhibitory activity with liver cancer cells (Hep3B). Moreover, the IC_50_ value of peucemycin on MRC-5 normal lung fibroblast cells 316 μM was much higher than that on A549 lung cancer cells (135.5 μM) (Fig. 4). Thus peucemycin showed modest activity with cancer cells. In contrast, doxorubicin has very toxic effects on the normal cells MRC-5 (IC_50_ 0.1 μM), human lung cancer cells A549 (IC_50_ 4.91 μM).^35^ The result indicated that peucemycin is less toxic than doxorubicin against the tested cell lines, which predicts the potential selectivity of peucemycin against the various cancer cells.

## Discussion

Natural products from microorganisms have been proven to be clinically important in human therapy as antifungal, antibacterial, antitumor, and anticancer agents. Two-thirds of medical antibiotics were isolated and identified from *Streptomyces* such as chloramphenicol, erythromycin, tetracycline, and streptomycin.^36–39^ The emergence and spread of antimicrobial resistance are serious problems for public health around the world;^40^ for example, *Streptococcus pneumoniae* and *Streptococcus pyogenes* are resistant to erythromycin;^41^*Staphylococcus aureus* and *Neisseria gonorrhoeae* are resistant to penicillin.^42,43^ Therefore, finding new bioactive compounds is an important strategy.

Previous studies have reported that environment stress such as temperature, pH, salinity, and elicitors affect the growth and productivity of microorganisms.^3,44^ Secondary metabolites including activity compounds were influenced by the culture temperature in *Pseudogymnoascus* sp., *Penicillium flavigenum*, and *Atradidymella* sp.^44^ Sadeghy and Hatami reported that *Streptomyces* sp. isolates C-26 and C-11 and there was a maximum metabolite production such as antifungal compounds when the culture condition was changed from 40 °C to 70 °C.^15^*Streptomyces* sp. RUPA-08PR increased the antibiotic production at 39 °C.^16^ Previously, *S. peucetius* showed the highest doxorubicin and daunorubicin production at 28 °C.^45^ In general, bioactive compounds are produced higher at room temperature; however, in this study, we demonstrated that a bioactive novel compound was successfully activated at a low temperature (18 °C) in *S. peucetius* (Fig. 1), which has never been achieved previously in this strain.

In this study, we described a structurally novel type I polyketide peucemycin. It is an unprecedented γ-pyrone macrocyclic with a cyclization γ-pyrone ring in the 14-membered macrolide. How cyclization occurs at the γ-pyrone ring in peucemycin is yet to be studied. However, onchidione from *Onchidium* sp. also has the same type of cyclization that contains two γ-pyrone rings.^46^ In natural products, pyrone macrolides have been reported present in alga: macrocyclic γ-pyrones from red alga *Phacelocarpus labillardieri*,^47^*Phacelocarpus peperocarpos*,^48^ neurymenolide A and B from the Fijian red alga *Neurymenia fraxinifolia*.^49^ This is the first time that a γ-pyrone macrolide has been reported from a bacteria.

In previous literature, macrolides have been used for infectious disease treatments instead of penicillin for patients allergic to penicillin. Erythromycin, the first macrolide, has a potential broad spectrum antimicrobial activity. Macrolide resistance mechanisms such as mutations of the antibiotic target (transition and transversion), decreased accumulation by enhanced efflux mediated, and hydrolysis degradation to the inactivation of macrolide were found.^50^ New macrolide ketolides exhibit greater activity with macrolide-resistant *streptococci*, *pneumococci*.^41,51^ Peucemycin shows a new type of chemical scaffold within the macrolide core, and this feature promises it to become a novel template for the design and development of new antibiotics. In this study, peucemycin shows antibacterial activities against some human pathogens (Fig. 3) and cytotoxicity against various cancer cell lines (Fig. 4). For further study, we will look for the pathway synthesis of this compound and characterize it in detail.

## Conclusion

In conclusion, we successfully activated and identified a new compound (peucemycin) by varying the culture temperature. The structure of peucemycin was elucidated *via* LC-MS and NMR. In addition, we examined the biological activity of peucemycin, which can be considered as a broader range antibacterial molecule and cytotoxicity against various cancer cell lines.

## Conflicts of interest

There are no conflicts to declare.

## Supplementary Material

RA-011-D0RA06099B-s001

## References

[cit1] Hindra, Pak P., Elliot M. A. (2010). J. Bacteriol..

[cit2] Bundale S., Begde D., Pillai D., Gangwani K., Nashikkar N., Kadam T., Upadhyay A. (2018). World J. Microbiol. Biotechnol..

[cit3] Sharma R., Jamwal V., Singh V. P., Wazir P., Awasthi P., Singh D., Gandhi S. G., Vishwakarma R. A., Chaubey A. (2017). J. Biotechnol..

[cit4] Blin K., Shaw S., Steinke K., Villebro R., Ziemert N., Lee S. Y., Medema M. H., Weber T. (2019). Nucleic Acids Res..

[cit5] Bednarz B., Kotowska M., Pawlik K. J. (2019). Appl. Microbiol. Biotechnol..

[cit6] Craney A., Ahmed S., Nodwell J. (2013). J. Antibiot..

[cit7] Du D., Katsuyama Y., Onaka H., Fujie M., Satoh N., Shin-ya K., Ohnishi Y. (2016). ChemBioChem.

[cit8] Nguyen H. T., Pham V. T. T., Nguyen C. T., Pokhrel A. R., Kim T., Kim D., Na K., Yamaguchi T., Sohng J. K. (2020). Appl. Microbiol. Biotechnol..

[cit9] Meng L. H., Zhang P., Li X. M., Wang B. G. (2015). Mar. Drugs.

[cit10] Meng L. H., Li X. M., Liu Y., Xu G. M., Wang B. G. (2017). RSC Adv..

[cit11] Meng L. H., Li X. M., Lv C. T., Huang C. G., Wang B. G. (2014). J. Nat. Prod..

[cit12] Lopez J. A. V., Nogawa T., Futamura Y., Shimizu T., Osada H. (2019). J. Antibiot..

[cit13] Wang Y., Lu Z., Sun K., Zhu W. (2011). Mar. Drugs.

[cit14] Auckloo B. N., Pan C., Akhter N., Wu B., Wu X., He S. (2017). Front. Microbiol..

[cit15] Sadeghy B., Hatami N. (2014). Arch. Phytopathol. Plant Prot..

[cit16] Ripa F. A., Nikkon F., Zaman S., Khondkar P. (2009). Mycobiology.

[cit17] Dhakal D., Lim S. K., Kim D. H., Kim B. G., Yamaguchi T., Sohng J. K. (2018). J. Biotechnol..

[cit18] Ghimire G. P., Oh T. J., Liou K., Sohng J. K. (2008). Mol. Cells.

[cit19] Kodani S., Komaki H., Suzuki M., Kobayakawa F., Hemmi H. (2015). BioMetals.

[cit20] Ghimire G. P., Koirala N., Sohng J. K. (2015). J. Microbiol. Biotechnol..

[cit21] Singh B., Oh T. J., Sohng J. K. (2009). J. Ind. Microbiol. Biotechnol..

[cit22] Thuan N. H., Dhakal D., Pokhrel A. R., Chu L. L., Van Pham T. T., Shrestha A., Sohng J. K. (2018). Appl. Microbiol. Biotechnol..

[cit23] Wilk W., Waldmann H., Kaiser M. (2009). Bioorg. Med. Chem..

[cit24] Singh S. B., Zink D. L., Dombrowski A. W., Dezeny G., Bills G. F., Felix J. P., Slaughter R. S., Goetz M. A. (2001). Org. Lett..

[cit25] Peng H., Ishida K., Sugimoto Y., Jenke-Kodama H., Hertweck C. (2019). Nat. Commun..

[cit26] Hayakawa Y., Saito J., Izawa M., Shin-Ya K. (2014). J. Antibiot..

[cit27] Liou S.-S., Shieh W.-L., Cheng T.-H., Won S.-J., Lin C.-N. (1993). J. Pharm. Pharmacol..

[cit28] Abou-Shoer M., Boettner F. E., Chang C. J., Cassady J. M. (1988). Phytochemistry.

[cit29] Arsic B., Barber J., Čikoš A., Mladenovic M., Stankovic N., Novak P. (2018). Int. J. Antimicrob. Agents.

[cit30] Kwon J. H. (2017). Infect. Dis..

[cit31] Kieser T. , Bibb M. J., Buttner M. J., Chater K. F. and Hopwood D. A., Practical Streptomyces Genetics, 2000

[cit32] Taher M., Susanti D., Rezali M. F., Zohri F. S. A., Ichwan S. J. A., Alkhamaiseh S. I., Ahmad F. (2012). Asian Pac. J. Trop. Med..

[cit33] Weinstein M. P. , M02 - Performance Standards for Antimicrobial Disk Susceptibility Tests, 13th edn, 2018

[cit34] Chaplin M. , Infrared Spectroscopy, http://www.ifsc.usp.br/∼lavfis2/BancoApostilasImagens/ApLuminescencia/InfraredSpectroscop1.pdf

[cit35] Benedeković G., Popsavin M., Kovačević I., Kojić V., Rodić M., Popsavin V. (2020). Eur. J. Med. Chem..

[cit36] Johnstone D. B., Waksman S. A. (1948). J. Bacteriol..

[cit37] Darken M. A., Berenson H., Shirk R. J., Sjolander N. O. (1960). Appl. Microbiol..

[cit38] Weber J. M., Wierman C. K., Hutchinson C. R. (1985). J. Bacteriol..

[cit39] Sekurova O. N., Zhang J., Kristiansen K. A., Zotchev S. B. (2016). Microb. Cell Fact..

[cit40] Cornaglia G., Ligozzi M., Mazzariol A., Valentini M., Orefici G., Fontana R. (1996). Emerging Infect. Dis..

[cit41] Descheemaeker P., Chapelle S., Lammens C., Hauchecorne M., Wijdooghe M., Vandamme P., Ieven M., Goossens H. (2000). J. Antimicrob. Chemother..

[cit42] Pantosti A., Sanchini A., Monaco M. (2007). Future Microbiol..

[cit43] Jones F., Cunningham E. J., Shockley T. E., Jackson J. H. (1985). Antimicrob. Agents Chemother..

[cit44] Yogabaanu U., Weber J. F. F., Convey P., Rizman-Idid M., Alias S. A. (2017). Polar Sci..

[cit45] Wang X., Tian X., Wu Y., Shen X., Yang S., Chen S. (2018). Prep. Biochem. Biotechnol..

[cit46] Carbone M., Gavagnin M., Mattia C. A., Lotti C., Castelluccio F., Pagano B., Mollo E., Guo Y. W., Cimino G. (2009). Tetrahedron.

[cit47] Kazlauskas R., Murphy P. T., Wells R. J., Blackman A. J. (1982). Aust. J. Chem..

[cit48] Murray L., Currie G., Capon R. J. (1995). Aust. J. Chem..

[cit49] Stout E. P., Hasemeyer A. P., Lane A. L., Davenport T. M., Engel S., Hay M. E., Fairchild C. R., Prudhomme J., Le Roch K., Aalbersberg W., Kubanek J. (2009). Org. Lett..

[cit50] Nakajima Y. (1999). J. Infect. Chemother..

[cit51] Lonks J. R., Goldmann D. A. (2005). Clin. Infect. Dis..

