# Supplementary material for: Identification and enhancing production of a novel macrolide compound in engineered Streptomyces peucetius
Source: RSC Adv. 2021 Jan 14;11(5):3168–73. doi: 10.1039/d0ra06099b (PMC8693821; doi:10.1039/d0ra06099b)
Supplement: RA-011-D0RA06099B-s001 [file RA-011-D0RA06099B-s001.pdf]

Electronic Supplementary Material (ESI) for RSC Advances.
This journal is © The Royal Society of Chemistry 2020

Supporting Information

Identification and enhancing production of a novel macrolide compound in engineered *Streptomyces peucetius*

Van Thuy Thi Pham[a], Hue Thi Nguyen[a], Chung Thanh Nguyen[a], Ye Seul Choi[a], Dipesh Dhakal[a], Tae-Su Kim[a], Hye Jin Jung[a,b], Tokutaro Yamaguchi[a], Jae Kyung Sohng[a,b,†]

[a]*Department of Life Science and Biochemical Engineering, SunMoon University, 70 Sunmoon-ro 221, Tangjeong-myeon, Asan-si, Chungnam 31460, Republic of Korea.*

[b]*Department of Pharmaceutical Engineering and Biotechnology, SunMoon University, 70 Sunmoon-ro 221, Tangjeong-myeon, Asan-si, Chungnam 31460, Republic of Korea.*

†Corresponding author:

Prof. Jae Kyung Sohng

Tel: +82(41)530-2246

Email: sohng@sunmoon.ac.kr

Fax: +82(41)530-8229

List of figures

Fig. S1 (a) The FT-IR spectrum of peucemycin 3

Fig. S1 (b) Fragment analysis of peucemycin 4

Fig. S2 (a) ^1H NMR (700 MHz, DMSO-*d6*) spectrum of peucemycin 5

Fig. S2 (b) ^{13}C NMR (176 MHz, DMSO-*d6*) spectrum of peucemycin 6

Fig. S2 (c) ^1H-^1H COSY (700 MHz, DMSO-*d6*) spectrum of peucemycin 7

Fig. S2 (d) ^1H-^1H ROESY (700 MHz, DMSO-*d6*) spectrum of peucemycin 8

Fig. S2 (e) HSQC (700 MHz, DMSO-*d6*) spectrum of peucemycin 9

Fig. S2 (f) HMBC (700 MHz, DMSO-*d6*) spectrum of peucemycin 10

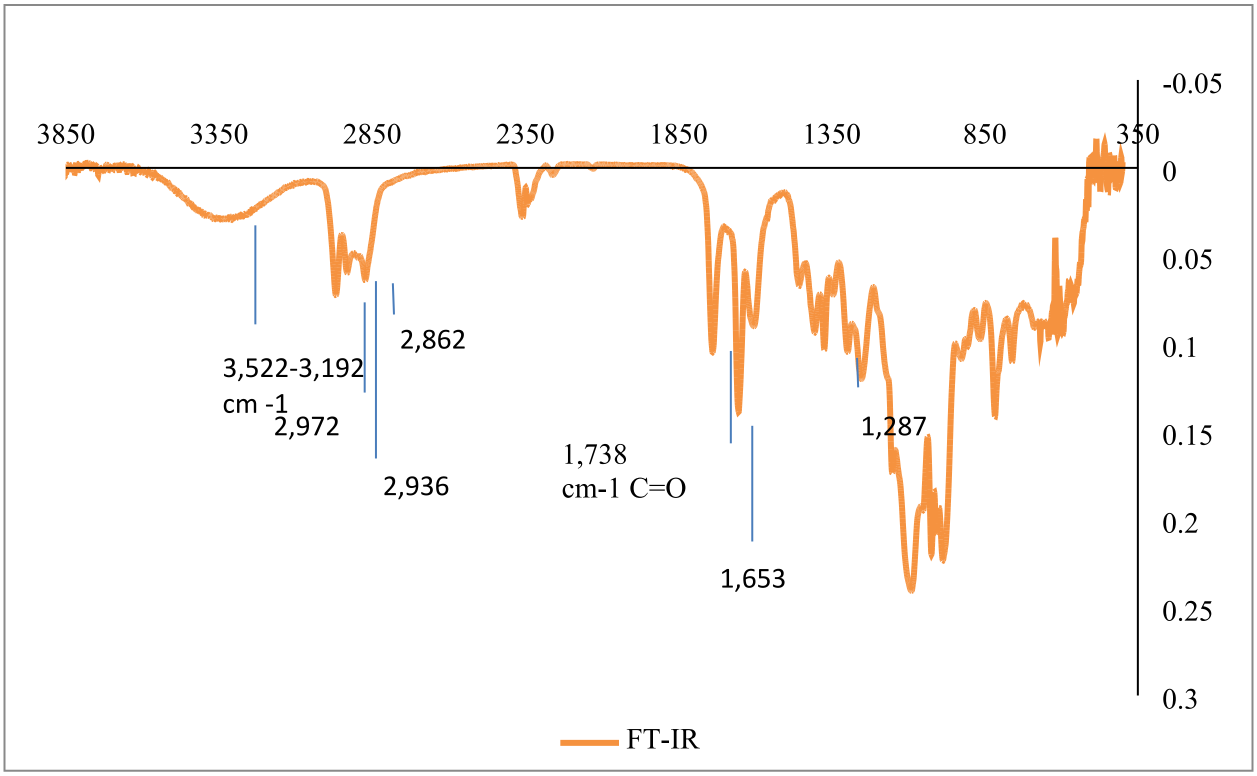

Fig. S1 (a) The FT-IR spectrum of peucemycin

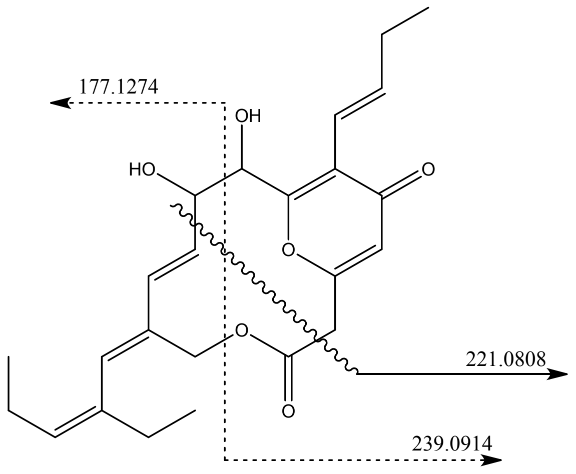

Chemical Formula: $C_{12}H_{17}O^+$
Exact Mass: 177.1274

Chemical Formula: $C_{12}H_{13}O_4^+$
Exact Mass: 221.0808

Chemical Formula: $C_{12}H_{15}O_5^+$
Exact Mass: 239.0914

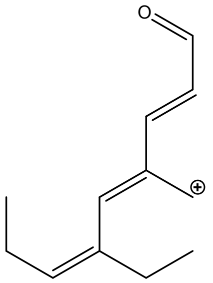

A

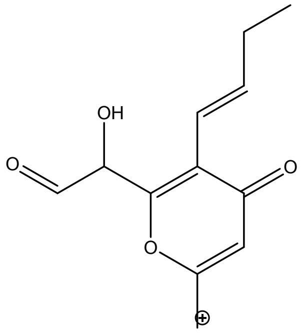

B

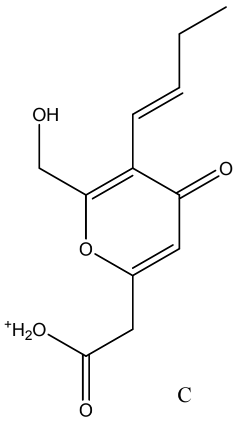

C

Fig. S1 (b) Fragment analysis of peucemycin

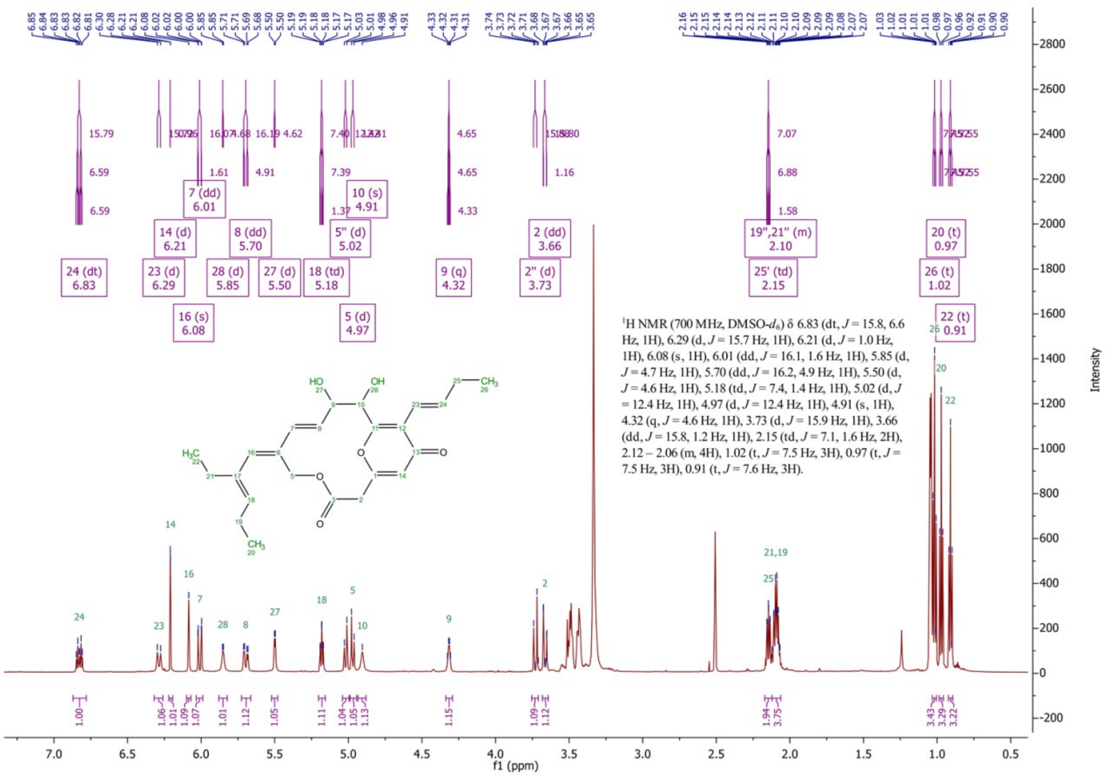

Fig. S2 (a) ^{1}H NMR (700 MHz, DMSO-*d6*) spectrum of peucemycin

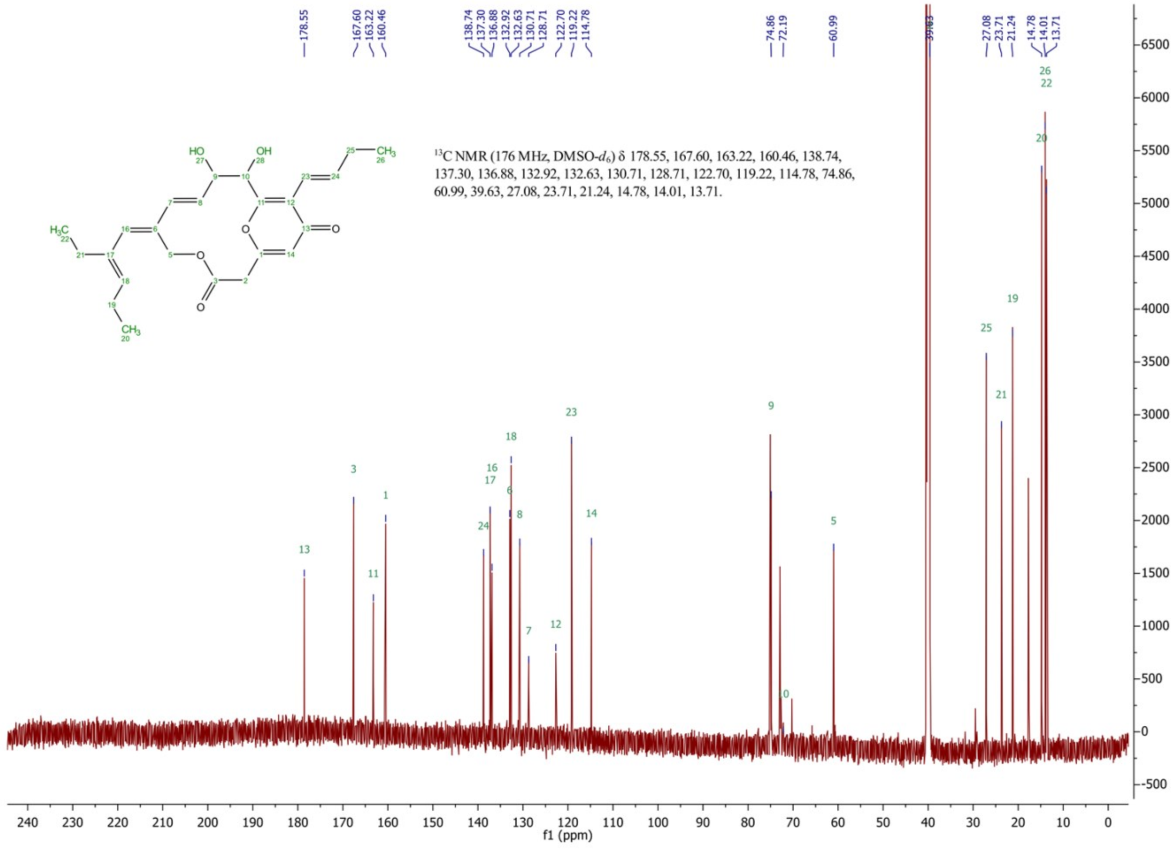

Fig. S2 (b) ^{13}C NMR (176 MHz, DMSO-*d6*) spectrum of peucemycin

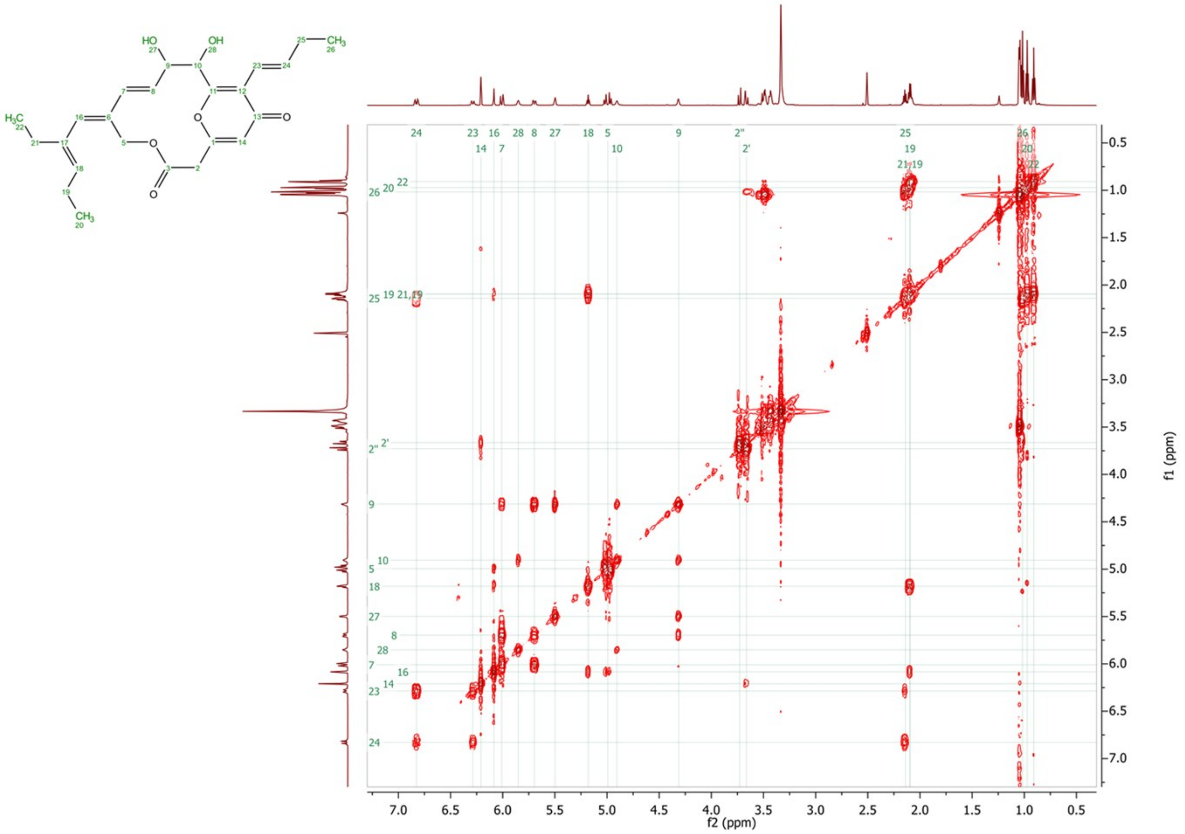

Fig. S2 (c) ^1H-^1H COSY (700 MHz, DMSO-*d6*) spectrum of peucemycin

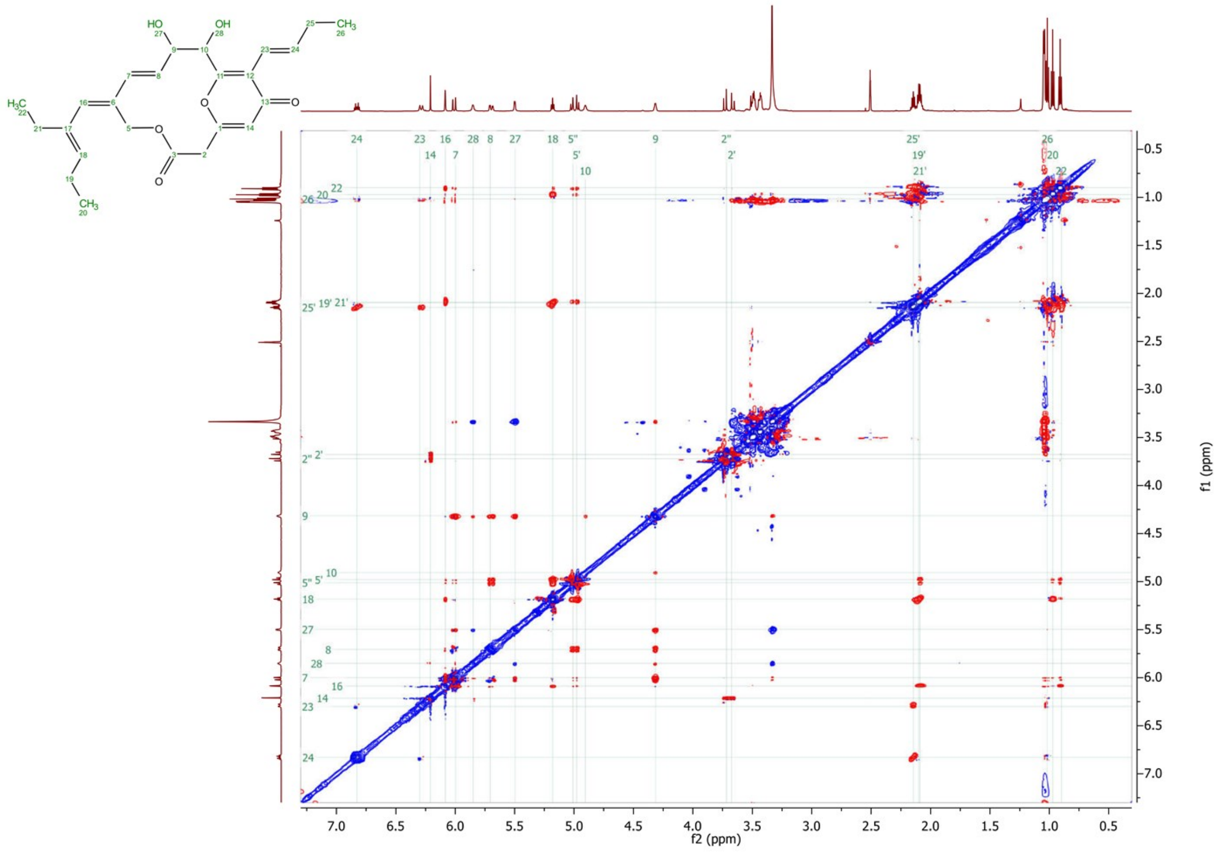

Fig. S2 (d) ^{1}H-^{1}H ROESY (700 MHz, DMSO-*d6*) spectrum of peucemycin

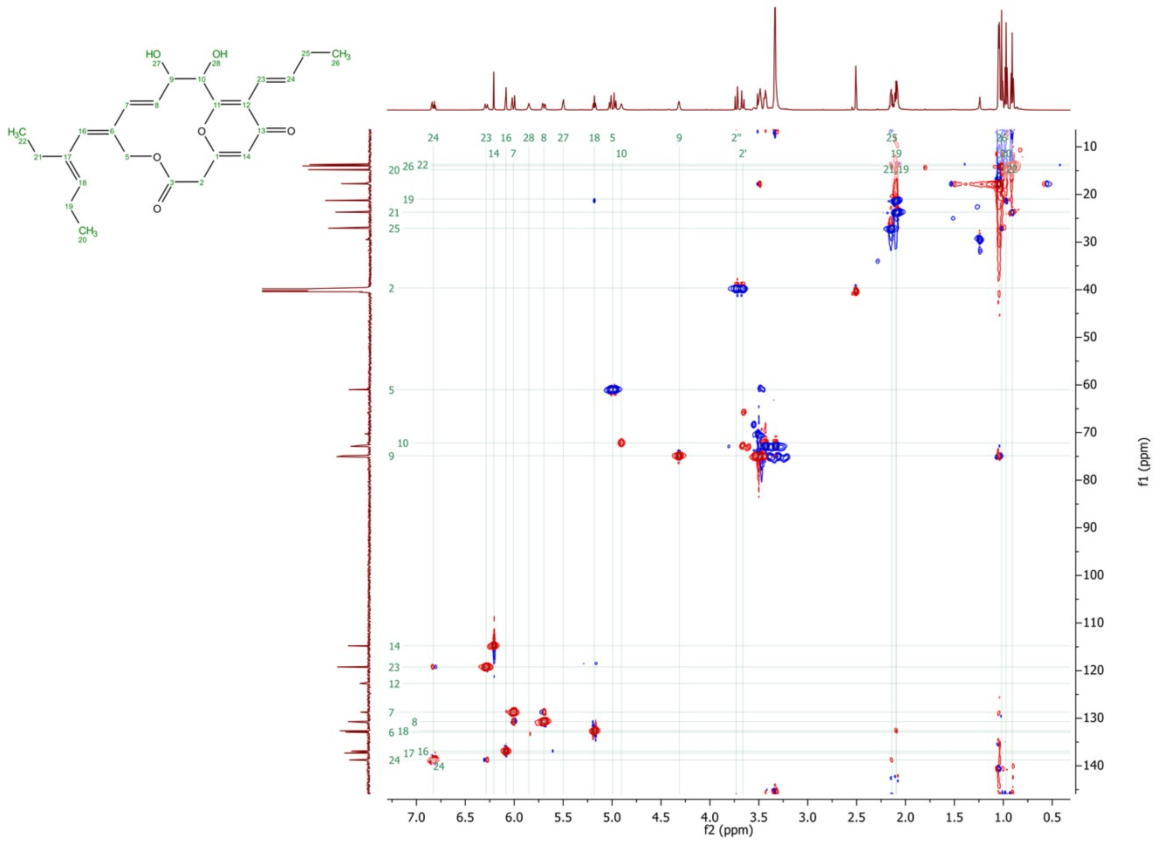

Fig. S2 (e) HSQC (700 MHz, DMSO-*d6*) **spectrum of peucemycin**

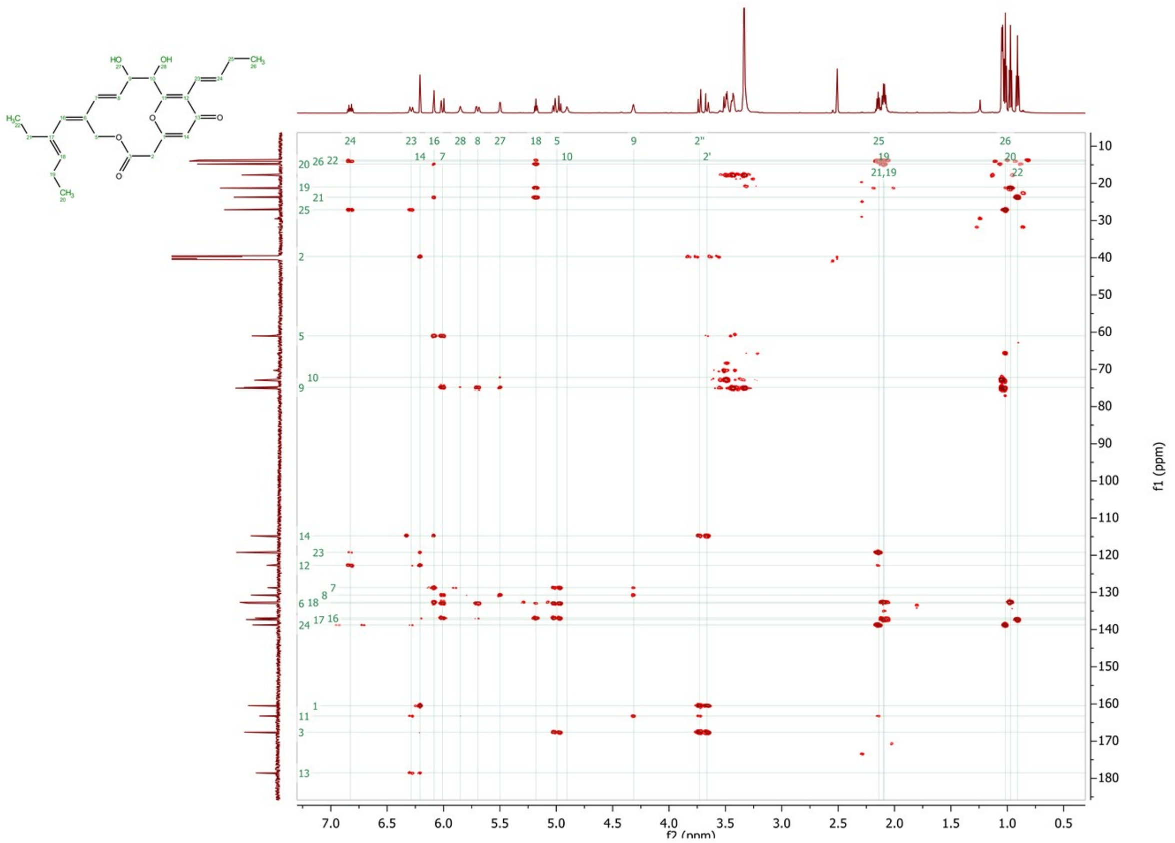

Fig. S2 (f) HMBC (700 MHz, DMSO-*d6*) spectrum of peucemycin
